# A study of fractal dimension as a quality indicator of hairtail (*Trichiurus haumela*) samples during frozen storage

**DOI:** 10.1038/s41598-018-33880-3

**Published:** 2018-11-07

**Authors:** Lanlan Luan, Yeshun Sun, Shiguo Chen, Chunhua Wu, Yaqin Hu

**Affiliations:** 1 National-Local Joint Engineering Laboratory of Intelligent Food Processing Technology and Equipment, Key Laboratory for Agro-Products Postharvest Handling of Ministry of Agriculture, Key Laboratory for Agro-Products Nutritional Evaluation of Ministry of Agriculture, Zhejiang Key Laboratory for Agro-Food Processing, Fuli Institute of Food Science, College of Biosystems Engineering and Food Science, Zhejiang University, Hangzhou, 310058 China; 2 Ocean Research Center of Zhoushan, Zhejiang University, Zhoushan, 316021 China; 3 College of Food Science, Fujian Agriculture and Forestry University, Fuzhou, 350002 China; 4 School of Mathematical Sciences, Zhejiang University, Hangzhou, 310058 China

## Abstract

Hairtail fish samples were stored at different freezing temperatures of −5 °C, −20 °C, −40 °C and −80 °C. To establish an effective quality evaluation approach for hairtail samples during frozen storage, fractal dimension was used to observe the porous microstructure that resulted from the ice crystal formation in frozen hairtail meat. The results showed that the initial value of fractal dimension of all the samples was 1.968. After frozen storage, the fractal dimension of samples at −5 °C, −20 °C, −40 °C and −80 °C decreased to 1.539, 1.682, 1.856 and 1.896, respectively. Traditional quality indicators such as water activity, color and textural properties (i.e., hardness, springiness and chewiness) also exhibited a similar decreasing trend, and the rate of change decreased with a decrease in storage temperature. The relationships were analyzed, and these traditional quality indicators were correlated with the fractal dimension with determined correlation coefficients within ±0.900. Moreover, based on the fractal dimension model, the first-order kinetic equation of fractal dimension variation during storage was ${{\bf{d}}}_{{\bf{t}}}={\bf{1.968}}\,{{\bf{e}}}^{{\boldsymbol{-}}{\bf{0.928}}{\bf{t}}{{\bf{e}}}^{{\boldsymbol{-}}{\bf{1431.396}}/{\bf{T}}}}$, which can be used to predict the shelf life of preserved hairtails at different storage temperatures. The results demonstrated fractal dimension was a novel and feasible method to evaluate the quality of hairtails in frozen storage.

## Introduction

Hairtail (*Trichiurus haumela*) is also called cutlassfish or ribbonfish, and it is a commercially important marine fish species in the eastern Pacific Ocean and Indian Ocean. Both ecologically and economically, the hairtail plays a significant role in supporting the most largest and valuable fishery in Asia with its abundant fish stocks^1,2^. The production and consumption of hairtail has recently been growing due to its delicious flavor and high nutritional value. However, hairtails are more perishable than other marine fish species because the meat contains highly unsaturated fatty acids that can negatively affect the color, flavor, texture and nutritional value^3,4^.

Freezing is a preferred technique for seafood preservation and is widely used from a domestic to an industrial scale. Freezing is much more popular than other preservation methods because enzymatic and microbial activity is lowered without the application of heat or preservatives^5^. Many studies have demonstrated that freezing also inevitably causes undesirable deterioration of fish quality, including lipid oxidation, water loss and undesirable flavor and texture, most of which can be generally assessed by traditional quality evaluation parameters, such as total volatile basic nitrogen (TVBN), thiobarbituric acid (TBA), the K value and a total plate count^6–9^. However, the impact of freezing on the microstructure of fish meat has rarely been studied. During the frozen storage of fish, ice crystallization and tissue dehydration can cause a honeycombed microstructure in the muscle tissue, which is a key factor that affects the ultimate quality of frozen food. Therefore, a new and more scientific quality evaluation approach based on microstructural variation is needed to improve the quality evaluation system for frozen food.

Mandelbrot proposed fractal dimension for this purpose, a geometric parameter that characterizes the space-filling capacity of a structure in space on a particular scale. Fractal dimension was recently used to quantify crystal networks of palm oil by image analysis and rheological measurements by Omar *et al*.^10^. Joardder *et al*. analyzed the correlation between the microstructure and porosity via the fractal dimension of dried foods^11^. Chen *et al*. established a fractal dimension model to analyze beef marbling standard images in China and the USA^12^.

The purpose of the present study is to investigate fractal dimension as an alternative advanced quality indicator to describe the porous microstructure of hairtails frozen at different temperatures. A correlation analysis with traditional quality indicators including water activity, color and textural properties was also done. Moreover, a viable method for shelf life prediction based on variations in fractal dimension during storage was established.

## Materials and Methods

### Chemicals

OCT (Optimum cutting temperature) embedding agent, ethanol, hematoxylin and eosin were bought from Sigma Chemical Co. (St. Louis, MO, USA). Dry ice was provided by Hangzhou Jingong Supplies Co., Ltd. (Hangzhou, Zhejiang, China). All of the chemicals used in this study were analytical reagent grade.

### Sample preparation

Fresh hairtails (length 70.0 ± 2.0 cm) were purchased from a local seafood market (Zhoushan, Zhejiang, China) and were immediately transported to the laboratory in an ice box. The hairtails were de-headed and gutted, and their tails were removed, followed by washing with sterile iced water. Then, the fish were drained and cut into blocks of approximately 6.0 cm in length. All of the fish blocks were sealed in the polyethylene bags and kept at temperatures of −5 ± 0.2 °C, −20 ± 1.5 °C, −40 ± 2.0 °C and −80 ± 2.0 °C, respectively. Samples stored at −5 °C were taken from the freezer for a quality analysis every 4 days, while the other three samples were analyzed every 7 days.

### Water activity analysis

The water activity (*Aw*) of hairtails frozen at different temperatures was determined with a meter (Aqualab 4TEV, Decagon, USA). Approximately 3 g of a hairtail sample was placed in the chamber, and the *Aw* was recorded when the value became constant.

### Color analysis

A color analysis of hairtail was carried out using a color meter (ColorFlex EZ, HunterLab, USA) immediately after defrosting, according to the previous method^13^. The equipment was calibrated with white and black standard calibration plate provided by the manufacture. The hairtail block was placed in a flat dish and the color measurement was repeated 10 times on different parts of the surface. In the L*a*b* system, L* denotes lightness on a scale from 0 to 100 from black to white; a* denotes (+) red or (−) green; and b* denotes (+) yellow or (−) blue^14^. Changes in the color coordinates (ΔL*Δa*Δb*) were calculated as ΔE*ab with the following formula^15^:

1 $${\Delta }{{E}}^{\ast }{ab}={[{({\Delta }{{L}}^{\ast })}^{2}+{({\Delta }{{a}}^{\ast })}^{2}+{({\Delta }{{b}}^{\ast })}^{2}]}^{1/2}.$$

### Textural analysis

A textural analysis was performed using a TA-XT2i texture analyzer according to the previous method^16^ with slight modifications. Measurements were made by pressing a flat-ended cylindrical probe (type p/5) into the blocks at a constant speed (1 mm/s) to achieve 50% compression relative to the block height. The TPA parameters including hardness, springiness and chewiness were determined. The textural attribute hardness is defined as the maximum force of the first compression. Springiness indicates how well a product physically springs back after it has been deformed during the first compression. Chewiness is the quantity that simulates the energy required to masticate a sample to a steady state for swallowing, and it is calculated as the product of hardness, cohesiveness and springiness^14^.

### Microscopic tissue imaging

Four pieces of approximately 5 mm in length were cut transversally to the muscle fiber from the center of frozen hairtail stored at each freezing temperature with a blade previously cooled to −20 °C. The entire operation was carried out in a walk-in freezer to ensure a perfect cold chain. Cryosections were obtained according to a previous method in the literature^17^ with some modifications. The frozen pieces were embedded in OCT embedding compound and submerged in dry ice until completely frozen. Each embedded sample was mounted on a chuck and placed in a CRYOSTAR NX50 (Thermo Scientific, USA) previously cooled to −20 °C. The surface of the hairtail specimen was trimmed and subsequently covered with an adhesive film (Cryofilm type 2 C(9), Section-Lab Co, Ltd., Japan) to support the frozen sections as previously described^18,19^. A14 µm thick section was carefully cut, fixed in 70% ethanol for 2 min, then stained with hematoxylin and eosin for later microscopic analysis.

All of the prepared sections were observed microscopically (UPH203i, Chongqing UOP Optoelectronic Technology Co., Ltd, China) fitted with a digital camera (JFMV-M1200C, Nanjing Yifei Technology Co., Ltd, China) at a magnification of 200×.

### Fractal dimension analysis

Fractal dimension values were obtained by the box-counting method^20^ using the MATLAB software. A microscopic color image was first converted into gray scale image, and processed with binarization. The binary image was divided into square sub-boxes of variable length denoted as *ε*. A sub-box filled by more than 50% of red tissue in a divided image was considered as valid. The number of the valid boxes in sections of different lengths was marked by *N* (*ε*). The fractal dimension d of the image can be obtained by the slope of the regression line on a plot of lg *N* (*ε*) against lg *ε*.

### Statistical analyses

All measurements were carried out in triplicate, and the data were expressed as the mean ± standard deviation. A t-test using SPSS 19 was used to test the significant differences of the means of the evaluation parameters, and a value of *P* < 0.05 was regarded as statistically significant.

## Results and Discussion

### Changes of water activity during frozen storage

**Figure 1:**
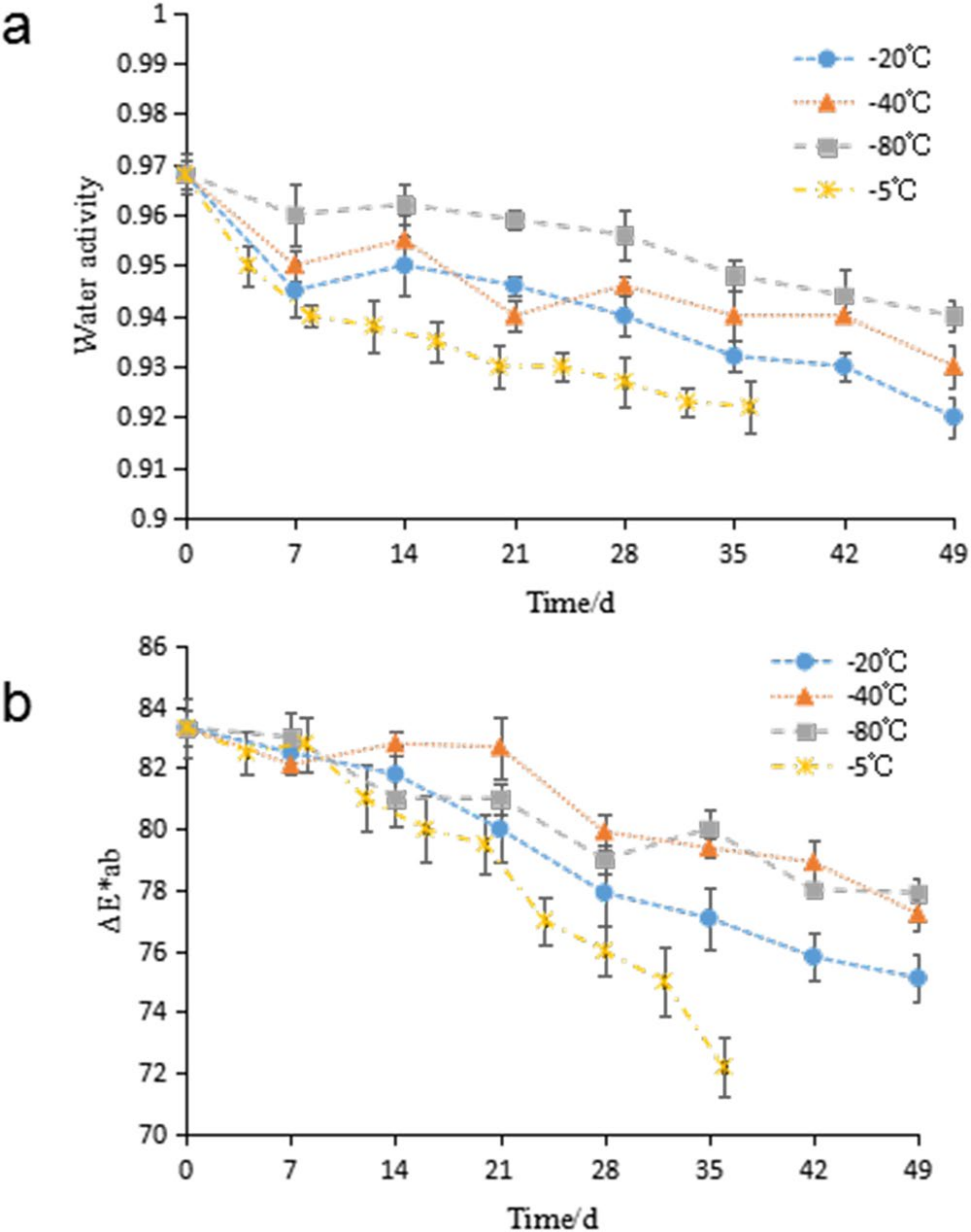
Changes in water activity (**a**) and ΔE*ab (**b**) of frozen hairtail samples stored at at −5 ^o^C (×), −20 ^o^C (●), −40 ^o^C (▲) and −80 ^o^C (■). The error bars indicate the standard deviation obtained from a total of three analysis.

The water activity of a food is a very important aspect of food preservation, and it is a key factor in fish spoilage and the growth of various microorganisms^21^. Figure 1a shows the variations in the water activity of hairtails stored at different temperatures. The initial value of water activity was 0.97, and it subsequently exhibited a decreasing trend throughout the storage period. The rate of decrease of water activity varied with the storage temperature. A higher storage temperature led to a greater significant decrease. The data showed that hairtail stored at −5 °C had significantly lower water activity than samples stored at the other temperatures.

A similar behavior of the water activity variation in fish preservation was found^22^. Fluid migration and ice crystallization during frozen storage could lead to a decrease in the water activity, as shown in this study. The changes also resulted in the development of a honeycombed microstructure in the muscle tissue^23^. Different rates of variation with temperature may be attributed to the formation of smaller ice crystals at lower temperature, which cause less tissue damage and less loosely bound water released from the cells^24^.

### Changes of color

All the effort to keep fish fresh is for consumption. Color assessment is the most direct and satisfactory way of evaluating the quality of freshness and the shelf life of fish in terms of consumer expectations^25^. The data for L*, a* and b* is not shown, but the changes in the total color difference ΔE*ab of hairtail fish meat during storage is shown in Fig. 1b. The figure shows that the initial ΔE*ab value of hairtails was 83.32. At the end of storage, the value respectively decreased to 72.20, 75.11, 77.21, and 77.90 in samples stored at −5 °C, −20 °C, −40 °C and −80 °C. It was also apparent that the hairtail muscle began to lose some of its original luster and color, and became dimmed as the storage time lengthened. Paola *et al*. published similar results^26^.

Since the changes of the L* value made the greatest contribution to the ΔE*ab^26^, the decrease of ΔE*ab may be due to the reduction of L*. The color analysis showed the L* value decreased during frozen storage, indicating a loss of the light color of the hairtail meat. The increase in the b* value may be explained by the presence of yellow pigments as a result of lipid oxidation. However, the original color was maintained in samples stored at −40 °C and −80 °C that had a lower rate of decrease of ΔE*ab, indicating that a lower temperature during frozen storage can inhibit the rate of enzymatic activity and lipid oxidation of hairtails.

### Changes of textural properties

**Figure 2:**
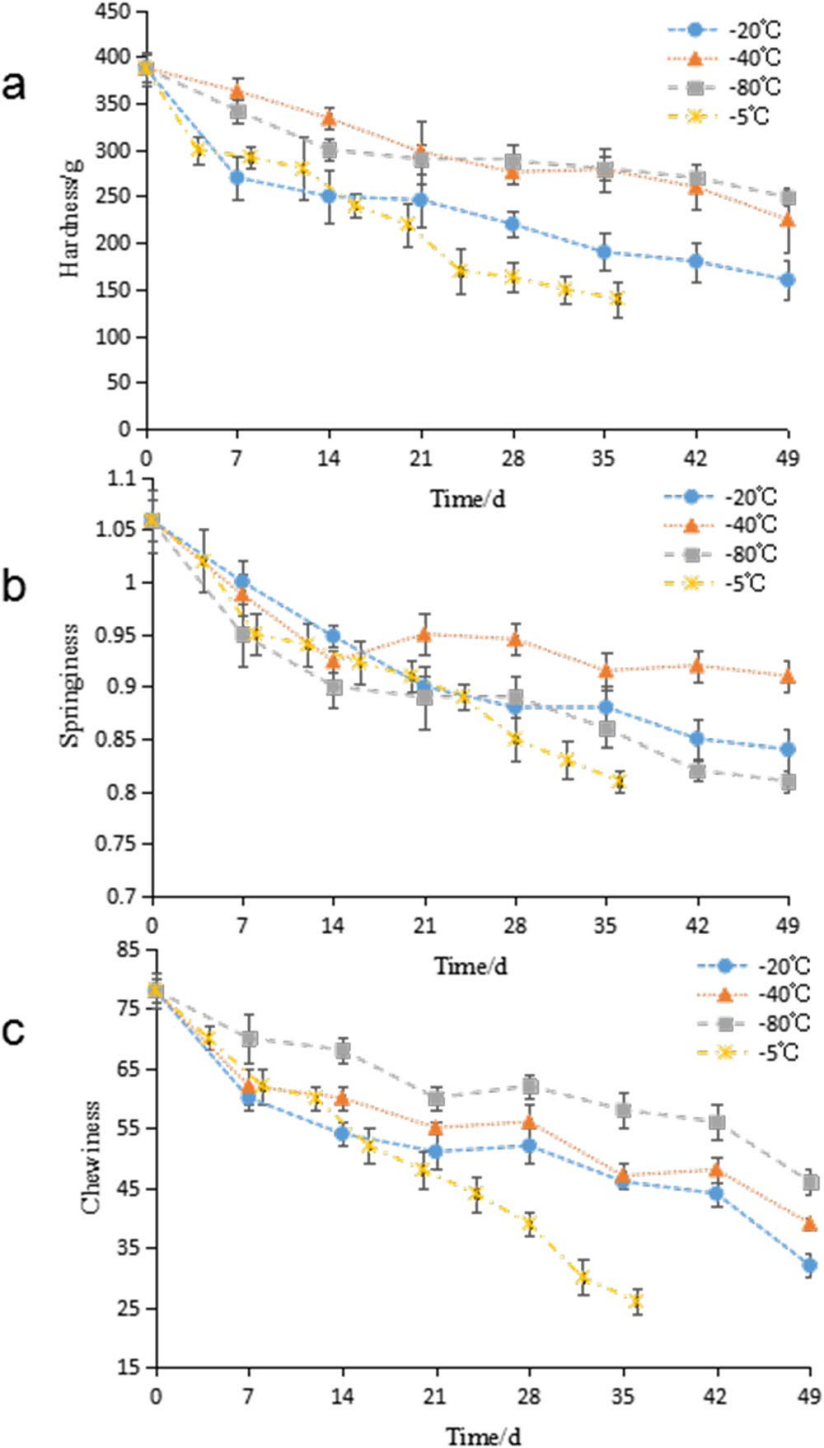
Changes in texture properties (**a** hardness, **b** springiness and **c** chewiness) of frozen hairtail samples stored at at −5 ^o^C (×), −20 ^o^C (●), −40 ^o^C (▲) and −80 ^o^C (■). The error bars indicate the standard deviation obtained from a total of three analysis.

The texture profile of hairtail meat is a valued quality indicator because it affects the sensory and functional properties of the fish^27^. Figure 2a shows that the samples stored at −5 °C had the lowest hardness after 36 days of storage, which had decreased by 63.9%, while the hardness of samples stored at −20 °C, −40 °C and −80 °C after 49 days was respectively reduced by 58.8%, 42.0%, and 35.8%. The hardness of fish may be associated with its muscle fibers. More closely packed muscle fibers confer a greater hardness value.

The springiness of the hairtail is shown in Fig. 2b. Springiness showed a decreasing trend similar to hardness as storage time lengthened. As expected, the springiness value was higher at lower temperatures except at −80 °C. A lower temperature preserves textural properties to a certain extent. However, some protein oxidation activity may occur in samples stored at −80 °C, which produces lower springiness.

Chewiness showed a trend similar to hardness, and was steadily reduced from 78 to 26, 32, 39, and 46 in samples stored at −5 °C, −20 °C, −40 °C, and −80 ^o^C, respectively (Fig. 2c). Protein oxidation and structural changes account for textural changes during frozen storage^27^. Paola *et al*. reported that the deterioration of the texture of fish flesh may be associated with various intrinsic and extrinsic factors, such as the loss of water from the muscle and the destruction of muscle tissue^28^.

### Changes of the tissue structure and the fractal dimension

**Figure 3:**
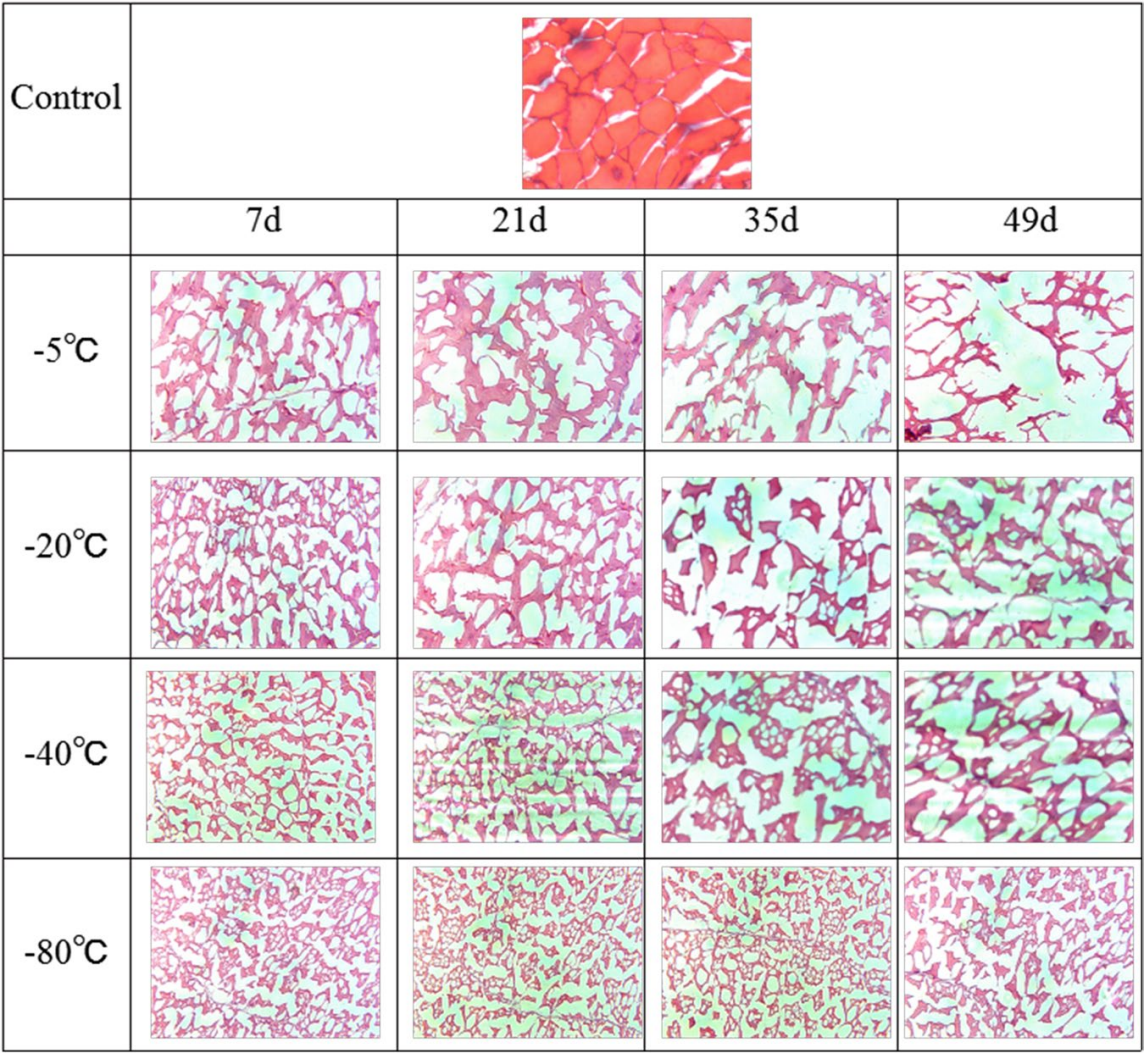
Changes in microstructure of frozen hairtail samples stored at at −5 ^o^C, −20 ^o^C, −40 ^o^C and −80 ^o^C.

Figure 3 shows the tissue structure of hairtails stored at different temperatures. The microstructure in the images of hairtail muscle comprises two parts: the red muscle tissues stained with H&E and the white space caused by ice crystals. Frozen hairtail muscle was compared with unfrozen tissue at 0 d as a control. The cross-section of a 0 d sample had a uniform distribution of regularly shaped fibers. The hairtail muscle fibers showed different degrees of departure from uniformity due to the ice crystal formation as the storage time increased. The images of samples under −5 ^o^C shows an increase of void spaces inside the frozen tissue during storage, compared to the initial samples. A similar trend is seen in samples under −20 ^o^C, −40 ^o^C and −80 ^o^C. This increasing trend coincides with that of Kaale *et al*.^29^, who also observed that the size of ice crystals formed in superchilled Atlantic salmon were significantly increased after 28 days of storage. The hairtail tissues initially showed only a few small voids, which grew into larger irregular pores with storage time, and this could be attributed to fluid migration caused by ice crystallization, and muscle fiber disarrangement caused by protein decomposition^29,30^.

Moreover, large and irregular ice crystals were observed in micrographs of hairtails stored at −5 ^o^C in Fig. 3. Clearly, most area was occupied with the cross-section of the ice crystals larger than the muscle fibers at 49 days storage, which implies that the muscle tissue of hairtails was seriously deformed. While the voids left by ice crystals in Fig. 3 at −80 ^o^C were still relatively small for 49 days of storage. Smaller and more regular ice crystals tended to form as the storage temperature decreased. Therefore, low temperature could inhibit structural changes and maintain hairtail tissue with more regular, smaller voids.

**Figure 4:**
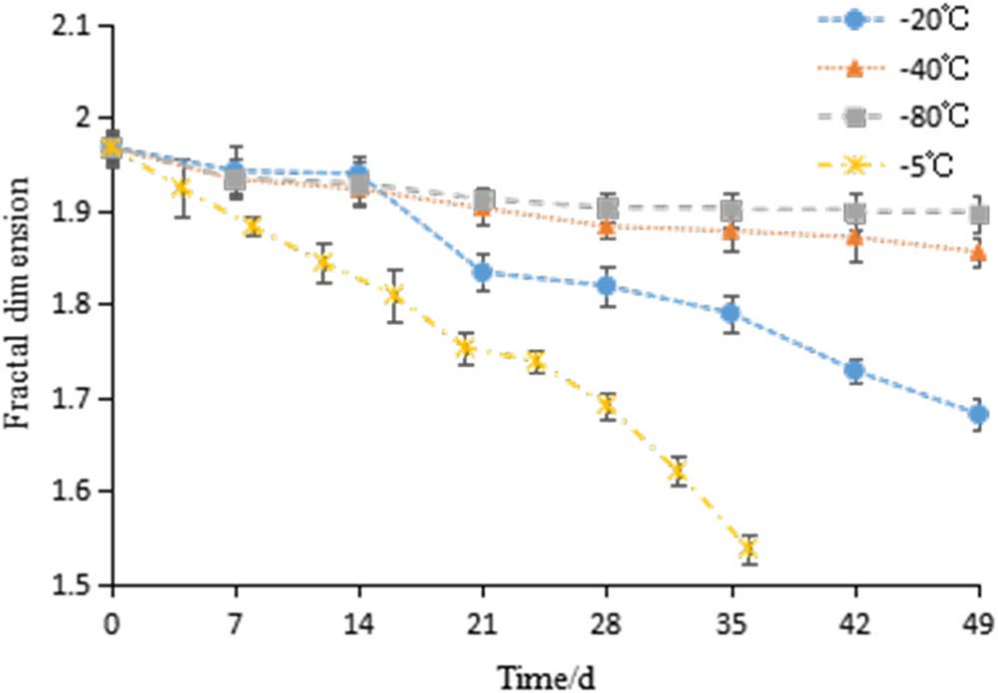
Changes in fractal dimension of frozen hairtail samples stored at at −5 ^o^C (×), −20 ^o^C (●), −40 ^o^C (▲) and −80 ^o^C (■). The error bars indicate the standard deviation obtained from a total of three analysis.

It was found that the porous microstructure that resulted from the ice crystals formation in frozen hairtail meat were scale free structure. Therefore, changes in the tissue structure can be quantitatively analyzed by measuring the fractal dimension, which is sensitive enough to distinguish tiny structural differences, size, or area fractions^31^, so it appropriately describes the uneven distribution of muscle fibers in hairtail tissue. The fractal dimension values of hairtail at various freezing temperatures and storage times are shown in Fig. 4. In general, the fractal dimension results exhibited a decreasing trend with frozen storage time, and the rate of decreasing was lower when stored at lower temperature. The initial value of fractal dimension of all the samples was 1.968. After frozen storage, the fractal dimension of samples at −5 ^o^C, −20 ^o^C, −40 ^o^C and −80 ^o^C decreased to 1.539, 1.682, 1.856 and 1.896, respectively. This result was in accordance with the findings reported by He *et al*.^32^, who found that fractal dimension of frozen tilapia tissues treated by tangerine peel extract decreased with increasing the temperature and storage time. A lower fractal dimension indicates a higher degree of irregularity of the microstructure caused by ice crystals. Large and irregular ice crystals may impart mechanical damage by physically rupturing cells, which may result in a reduction in fractal dimension, an increase in drip loss, protein denaturation and other quality changes related to the damage of the cell structure^29^.

### Relationship between fractal dimension and traditional quality parameters

The Pearson correlation coefficient is widely used to determine the degree of linear dependence between two variables^33^. Table 1 shows the Pearson correlation coefficients between fractal dimension and various traditional quality parameters, including the water activity, ΔE*ab, hardness, springiness and chewiness. All of these parameters were positively correlated with the fractal dimension, and high correlation coefficients (0.909 < *R*^2^ < 0.993) were obtained. The results suggested that fractal dimension can be applied as a reliable quality indicator to quantitatively assess variations in quality parameters.

**Table 1:** Pearson correlation coefficients of fractal dimension in relation to water activity, ΔE*ab, hardness, springiness and chewiness.

Temperature/^o^C	Water activity	ΔE*ab	Hardness	Springiness	Chewiness
−5	0.960	0.963	0.959	0.916	0.993
−20	0.941	0.982	0.975	0.924	0.972
−40	0.961	0.909	0.972	0.969	0.928
−80	0.964	0.979	0.927	0.919	0.931

### Prediction model of shelf-life by fractal dimension

The shelf-life of the hairtail was predicted by a first-order kinetics model using the fractal dimension of the hairtail tissue as the key quality factor. The model can be expressed as Eq. 2.

2 $${d}_{t}={d}_{0}\,{e}^{{k}_{T}t}$$

where *t* is the storage time (d), *d*_*t*_ is the fractal dimension value at a storage time *t*, *d*_0_ is the initial value of the fractal dimension, and *k*_*T*_ is the rate constant at a storage temperature *T*.

By using the storage time *t* as the X axis, and the value of ln (*d*_*t*_/*d*_0_) of the hairtails stored at different temperature as the Y axis, the slopes of the regression line (*k*_*T*_) at 268 K, 253 K, 233 K and 193 K were obtained as −0.0063, −0.0033, −0.0011 and −0.0007, respectively. It indicated that the higher the storage temperature, the greater the absolute value of *k*_*T*_.

Additionally, the relationship between the rate constant *k*_*T*_ and the storage time *T* can be shown by the Arrhenius equation as follows^34^:

3 $${k}_{T}={k}_{0}{e}^{-{E}_{a}/(RT)}$$

where *k*_0_ is the pre-exponential factor (d^−1^), *E*_*a*_ is the activation energy (kJ/mol), and *R* is the gas constant (8.314 J/mol K). *T*, *k*_*T*_, *k*_0_ and *E*_*a*_ are constants associated with the physical nature of the reaction system.

Incorporating Eq. 2 with Eq. 3, a global equation can be formulated:

4 $${d}_{t}={d}_{0}\,{e}^{{k}_{0}t{e}^{-{E}_{a}/(RT)}}$$

Plotting ln (−*k*_*T*_) with 1/*T*, the fitting line was obtained as y = −1431.300 × −0.075 (*R*^2^ = 0.991). So the activation energy *E*_*a*_ of the samples was calculated as 11900.400 kJ/mol, and the pre-exponential factor *k*_0_ as −0.928 d^−1^. Accordingly, the first-order kinetics equation of fractal dimension was obtained as follows:

5 $${d}_{t}=1.968\,{e}^{-0.928t{e}^{-1431.369/T}}$$

This equation indicated that there is some kind of exponential relationship between the final fractal dimension of frozen hairtails and storage time *t* and storage temperature *T*. Higher storage temperature *T* will result a lower shel-life *t* of frozen hairtails.

In addition, the peak fractal dimension measured in spoiled hairtail was 1.530 ± 0.013. Table 2 lists the shelf-life results. The data agreed with similar studies^35,36^. The errors were within ±8.61%, so the accuracy of the method is acceptable.

**Table 2:** Predicted and measured shelf-life of frozen hairtails stored at different temperatures.

Temperature/^o^C	Predicted value/d	Measured value/d	Error/%
−5	56.66	62.00	−8.61
−20	77.65	74.00	4.93
−40	126.42	130.00	−2.75
−80	450.84	429.00	5.09

## Conclusion

Structural and chemical changes of frozen hairtail tissues were analyzed in the present study. The fractal dimension, which quantifies the porous structure formed in tissue samples, as well as water activity, color, hardness, springiness and chewiness all decreased during frozen storage. The rate of change of these parameters decreased with a decrease in the storage temperature. Variations of fractal dimension during storage were significantly correlated with the traditional quality parameters. Therefore, the fractal dimension of hairtail tissue could be used as an effective indicator to characterize changes in the quality of hairtail. Furthermore, the shelf life of preserved hairtails under different storage temperatures can be accurately predicted with the first-order kinetic equation ${d}_{t}=1.968\,{e}^{-0.928t{e}^{-1431.369/T}}$. Fractal dimension is a novel quality indicator that will provide new insights in the field of food preservation.

## Data Availability

The datasets generated during and/or analysed during the current study are available from the corresponding author on reasonable request.
